# Biomarkers for personalised prevention of chronic diseases: a common protocol for three rapid scoping reviews

**DOI:** 10.1186/s13643-024-02554-9

**Published:** 2024-06-01

**Authors:** E Plans-Beriso, C Babb-de-Villiers, D Petrova, C Barahona-López, P Diez-Echave, O R Hernández, N F Fernández-Martínez, H Turner, E García-Ovejero, O Craciun, P Fernández-Navarro, N Fernández-Larrea, E García-Esquinas, I Kuhn, V Jiménez-Planet, V Moreno, F Rodríguez-Artalejo, M J Sánchez, M Pollan-Santamaria, L Blackburn, M Kroese, B Pérez-Gómez

**Affiliations:** 1 Department of Epidemiology of Chronic Diseases, National Centre for Epidemiology, Instituto de Salud Carlos III, Madrid, Spain; 2 CIBER of Epidemiology and Public Health (CIBERESP), Madrid, Spain; 3 PHG Foundation, University of Cambridge, Cambridge, UK; 4 Instituto de Investigación Biosanitaria Ibs. GRANADA, Granada, Spain; 5 Escuela Andaluza de Salud Pública (EASP), Granada, Spain; 6 Cambridge University Medical Library, Cambridge, UK; 7 National Library of Health Sciences, Instituto de Salud Carlos III, Madrid, Spain; 8 Oncology Data Analytics Program, Catalan Institute of Oncology (ICO), L’Hospitalet de Llobregat, Barcelona, 08908 Spain; 9 Colorectal Cancer Group, ONCOBELL Program, Institut de Recerca Biomedica de Bellvitge (IDIBELL), L’Hospitalet de Llobregat, Barcelona, 08908 Spain; 10 Department of Preventive Medicine and Public Health, Universidad Autónoma de Madrid, Madrid, Spain; 11 IMDEA-Food Institute, CEI UAM+CSIC, Madrid, Spain

**Keywords:** Personalised prevention, Precision Medicine, Precision prevention, Biomarkers, Cancer, Neoplasm, Cardiovascular diseases, Neurodegenerative diseases, Chronic diseases

## Abstract

**Introduction:**

Personalised prevention aims to delay or avoid disease occurrence, progression, and recurrence of disease through the adoption of targeted interventions that consider the individual biological, including genetic data, environmental and behavioural characteristics, as well as the socio-cultural context. This protocol summarises the main features of a rapid scoping review to show the research landscape on biomarkers or a combination of biomarkers that may help to better identify subgroups of individuals with different risks of developing specific diseases in which specific preventive strategies could have an impact on clinical outcomes.

This review is part of the “Personalised Prevention Roadmap for the future HEalThcare” (PROPHET) project, which seeks to highlight the gaps in current personalised preventive approaches, in order to develop a Strategic Research and Innovation Agenda for the European Union.

**Objective:**

To systematically map and review the evidence of biomarkers that are available or under development in cancer, cardiovascular and neurodegenerative diseases that are or can be used for personalised prevention in the general population, in clinical or public health settings.

**Methods:**

Three rapid scoping reviews are being conducted in parallel (February–June 2023), based on a common framework with some adjustments to suit each specific condition (cancer, cardiovascular or neurodegenerative diseases). Medline and Embase will be searched to identify publications between 2020 and 2023. To shorten the time frames, 10% of the papers will undergo screening by two reviewers and only English-language papers will be considered. The following information will be extracted by two reviewers from all the publications selected for inclusion: source type, citation details, country, inclusion/exclusion criteria (population, concept, context, type of evidence source), study methods, and key findings relevant to the review question/s. The selection criteria and the extraction sheet will be pre-tested. Relevant biomarkers for risk prediction and stratification will be recorded. Results will be presented graphically using an evidence map.

**Inclusion criteria:**

Population: general adult populations or adults from specific pre-defined high-risk subgroups; concept: all studies focusing on molecular, cellular, physiological, or imaging biomarkers used for individualised primary or secondary prevention of the diseases of interest; context: clinical or public health settings.

**Systematic review registration:**

https://doi.org/10.17605/OSF.IO/7JRWD (OSF registration DOI).

**Supplementary Information:**

The online version contains supplementary material available at 10.1186/s13643-024-02554-9.

## Background

In recent years, innovative health research has moved quickly towards a new paradigm. The ability to analyse and process previously unseen sources and amounts of data, e.g. environmental, clinical, socio-demographic, epidemiological, and ‘omics-derived, has created opportunities in the understanding and prevention of chronic diseases, and in the development of targeted therapies that can cure them. This paradigm has come to be known as “personalised medicine”. According to the European Council Conclusion on personalised medicine for patients (2015/C 421/03), this term defines a medical model which involves characterisation of individuals’ genotypes, phenotypes and lifestyle and environmental exposures (e.g. molecular profiling, medical imaging, lifestyle and environmental data) for tailoring the right therapeutic strategy for the right person at the right time, and/or to determine the predisposition to disease and/or to deliver timely and targeted prevention [1, 2]. In many cases, these personalised health strategies have been based on advances in fields such as molecular biology, genetic engineering, bioinformatics, diagnostic imaging and new’omics technologies, which have made it possible to identify biomarkers that have been used to design and adapt therapies to specific patients or groups of patients [2]. A biomarker is defined as a substance, structure, characteristic, or process that can be objectively quantified as an indicator of typical biological functions, disease processes, or biological reactions to exposure [3, 4].

Adopting a public health perspective within this framework, one of the most relevant areas that would benefit from these new opportunities is the personalisation of disease prevention. *Personalised prevention* aims to delay or avoid the occurrence, progression and recurrence of disease by adopting targeted interventions that take into account biological information, environmental and behavioural characteristics, and the socio-economic and cultural context of individuals. These interventions should be timely, effective and equitable in order to maintain the best possible balance in lifetime health trajectory [5].

Among the main diseases that merit specific attention are chronic noncommunicable diseases, due to their incidence, their mortality or disability-adjusted life years [6–9]. Within the European Union (EU), in 2021, one-third of adults reported suffering from a chronic condition [10]. In addition, in 2019, the leading causes of mortality were cardiovascular disease (CVD) (35%), cancer (26%), respiratory disease (8%), and Alzheimer's disease (5%) [11]. For all of the above, in 2019, the PRECeDI consortium recommended the identification of biomarkers that could be used for the prevention of chronic diseases to integrate personalised medicine in the field of chronicity. This will support the goal of stratifying populations by indicating an individuals’ risk or resistance to disease and their potential response to drugs, guiding primary, secondary and tertiary preventive interventions [12]; understanding primary prevention as measures taken to prevent the occurrence of a disease before it occurs, secondary prevention as actions aimed at early detection, and tertiary prevention as interventions to prevent complications and improve quality of life in individuals already affected by a disease [4].

The “Personalised Prevention roadmap for the future HEalThcare” (PROPHET) project, funded by the European Union’s Horizon Europe research and innovation program and linked to ICPerMed, seeks to assess the effectiveness, clinical utility, and existing gaps in current personalised preventive approaches, as well as their potential to be implemented in healthcare settings. It also aims to develop a Strategy Research and Innovation Agenda (SRIA) for the European Union. This protocol corresponds to one of the first steps in the PROPHET, namely a review that aims to map the evidence and highlight the evidence gaps in research or the use of biomarkers in personalised prevention in the general adult population, as well as their integration with digital technologies, including wearable devices, accelerometers, and other appliances utilised for measuring physical and physiological functions. These biomarkers may be already available or currently under development in the fields of cancer, CVD, and neurodegenerative diseases.

There is already a significant body of knowledge about primary and secondary prevention strategies for these diseases. For example, hypercholesterolemia or dyslipidaemia, hypertension, smoking, diabetes mellitus and obesity or levels of physical activity are known risk factors for CVD [6, 13] and neurodegenerative diseases [14–16]; for cancer, a summary of lifestyle preventive actions with good evidence is included in the European code against cancer [17]. The question is whether there is any biomarker or combination of biomarkers that can help to better identify subgroups of individuals with different risks of developing a particular disease, in which specific preventive strategies could have an impact on clinical outcomes. Our aim in this context is to show the available research in this field.

Given the context and time constraints, the rapid scoping review design is the most appropriate method for providing landscape knowledge [18] and provide summary maps, such as Campbell evidence and gap map [19]. Here, we present the protocol that will be used to elaborate three rapid scoping reviews and evidence maps of research on biomarkers investigated in relation to primary or secondary prevention of cancer, cardiovascular and neurodegenerative diseases, respectively. The results of these three rapid scoping reviews will contribute to inform the development of the PROPHET SRIA, which will guide the future policy for research in this field in the EU.

## Review question

What biomarkers are being investigated in the context of personalised primary and secondary prevention of cancer, CVD and neurodegenerative diseases in the general adult population in clinical or public health settings?

## Methods

Three rapid scoping reviews are being conducted between February and June 2023, in parallel, one for each disease group included (cancer, CVD and neurodegenerative diseases), using a common framework and specifying the adaptations to each disease group in search terms, data extraction and representation of results.

This research protocol, designed according to Joanna Briggs Institute (JBI) and Preferred Reporting Items for Systematic Reviews and Meta-Analyses extension for Scoping Reviews (PRISMA-ScR) Checklist [20–22] was uploaded to the Open Science Framework for public consultation [23], with registration DOI https://doi.org/10.17605/OSF.IO/7JRWD. The protocol was also reviewed by experts in the field, after which modifications were incorporated.

### Eligibility criteria

Following the PCC (population, concept and context) model [21, 22], the included studies will meet the following eligibility criteria (Table 1):

**Table 1:** Eligibility criteria^a^

	Inclusion	Exclusion
Population	✔ Adult general population (> 18 years) ✔ Apart from the general population, we have included relevant specific subgroups of high-risk adults depending on the group of diseases to which they belong: - For all diseases: Smoking, alcohol consumption, diabetes, obesity, family history - For cancer: HPV, liver cirrhosis, hepatitis virus (HVB, HVC, HVD), *Helicobacter pylori*, HIV infection - For CVD: arterial hypertension, hypercholesterolemia or dyslipidaemia, chronic kidney disease - For neurodegenerative diseases: hypertension, hypercholesterolemia or dyslipidaemia, APOE genotype, hearing impairment ✔ People who have already had the disease of interest, have it or in whom there is a proxy for the disease ✔ Any country of origin	× Pregnancy
Concept	✔ Studies with a focus on molecular, cellular, physiological, and imaging biomarkers^b^ used for individualised primary or secondary prevention of the following diseases^c^: ◦ Cancer: breast, lung, prostate, colon, bladder, rectum, pancreas, liver, stomach, corpus, and cervix uteri ◦ CVD: ischemic heart disease, stroke, cardiomyopathy and myocarditis, atrial fibrillation and atrial flutter, aortic aneurysm, nonrheumatic valvular heart disease (calcific aortic valve disease, degenerative mitral valve disease), peripheral artery disease ◦ Neurodegenerative diseases: amyotrophic lateral sclerosis, Parkinson’s disease, Alzheimer’s disease, frontotemporal dementia, vascular dementia, Lewy body disease and multiple sclerosis ✔ In primary prevention: Biomarkers should stratify individuals into groups ✔ In secondary prevention: Biomarkers should improve the screening or early detection: ◦ stratifying different risk groups ◦ improving the sensitivity, specificity, and time of diagnosis for new subgroups of individuals ✔ Studies with some risk stratification related to the early detection (not to the evolution of the disease)	× Studies with a focus on diagnostic biomarkers are not used for secondary prevention × Studies with a focus on biomarkers used for prognosis except for prostate cancer × Any infectious aetiology for cardiovascular diseases included in this scoping review × Studies focusing only on well-established biomarkers used for the diagnosis of any of the diseases, infections, and conditions in the specific high-risk population groups
Context	✔ Clinical or public health settings ✔ English language only ✔ Any geographical setting	× Studies published before 2020
Type of evidence	✔ Reviews: Umbrella review, systematic review, meta-analysis, scoping review ✔ Experimental and quasi-experimental study designs: Randomised controlled trials, non-randomised controlled trials, before and after studies, interrupted time-series studies ✔ Analytical observational study designs: Prospective and retrospective cohort studies, case–control studies, and analytical cross-sectional studies ✔ Descriptive observational study designs: Descriptive cross-sectional studies	× Editorials and opinion pieces × Narrative reviews × Protocols × Qualitative study designs. Delphi studies × Conference abstracts, conference reports × Clinical practice guidelines × Basic research (i.e. laboratory research in animals, human tissues, and cell lines) × Data simulation or modelling studies × Case reports and case series
Sources	✔ MEDLINE via Ovid ✔ Embase via Ovid ✔ Embase preprints via Ovid	

Terms used in this table have been compiled in a glossary (Additional file 1)

^a^These criteria eventually could be adapted after the title and abstract screening of the first 10% of the papers

^b^Those biomarkers used to define the specific high-risk groups (i.e. glucose levels for diabetes, lipid levels for hypercholesterolemia, BMI for obesity) will not be considered alone, unless they are complemented by new biomarkers or personal information (see Additional file 2). Imaging tests will be considered as a biomarker

^c^Research on intermediary disease outcomes (e.g. cognitive decline stands for dementia, lung nodule stands for lung cancer) will also be included if there is a clear association with the outcomes of interest and the paper explicitly declares such association from previous evidence

#### Rationale for performing a rapid scoping review

As explained above, these scoping reviews are intended to be one of the first materials produced in the PROPHET project, so that they can inform the first draft of the SRIA. Therefore, according to the planned timetable, the reviews should be completed in only 4 months. Thus, following recommendations from the Cochrane Rapid Review Methods Group [24] and taking into account the large number of records expected to be assessed, according to the preliminary searches, and in order to meet these deadlines, specific restrictions were defined for the search—limited to a 3-year period (2020–2023), in English only, and using only MEDLINE and EMBASE as possible sources—and it was decided that the title-abstract and full-text screening phase would be carried out by a single reviewer, after an initial training phase with 10% of the records assessed by two reviewers to ensure concordance between team members. This percentage could be increased if necessary.

#### Rationale for population selection

These rapid scoping reviews are focused on the general adult population. In addition, they give attention to studies conducted among populations that present specific risk factors relevant to the selected diseases or that include these factors among those considered in the study.

For cancer, these risk (or preventive) factors include smoking [25], obesity [26], diabetes [27–29], *Helicobacter pylori* infection/colonisation [30], human papillomavirus (HPV) infection [30], human immunodeficiency virus (HIV) infection [30], alcohol consumption [31], liver cirrhosis and viral (HVB, HVC, HVD) hepatitis [32].

For CVD, we include hypercholesterolemia or dyslipidaemia, arterial hypertension, smoking, diabetes mellitus, chronic kidney disease, hyperglycaemia and obesity [6, 13].

Risk groups for neurodegenerative diseases were defined based on the following risk factors: obesity [15, 33], arterial hypertension [15, 33–35], diabetes mellitus [15, 33–35], dyslipidaemia [33], alcohol consumption [36, 37] and smoking [15, 16, 33, 34].

After the general search, only relevant and/or disease-specific subpopulations will be used for each specific disease. On the other hand, pregnancy is an exclusion criterion, as the very specific characteristics of this population group would require a specific review.

#### Rationale for disease selection

The search is limited to diseases with high morbidity and mortality within each of the three disease groups:

##### Cancer type

Due to time constraints, we only evaluate those malignant neoplasms with the greatest mortality and incidence rates in Europe, which according to the European Cancer Information System [38] are breast, prostate, colorectum, lung, bladder, pancreas, liver, stomach, kidney, and corpus uteri. Additionally, cervix uteri and liver cancers will also be included due to their preventable nature and/or the existence of public health screening programs [30, 31].

##### CVD

We evaluate the following main causes of deaths: ischemic heart disease (49.2% of all CVD deaths), stroke (35.2%) (this includes ischemic stroke, intracerebral haemorrhage and subarachnoid haemorrhage), hypertensive heart disease (6.2%), cardiomyopathy and myocarditis (1.8%), atrial fibrillation and flutter (1.7%), rheumatic heart disease (1.6%), non-rheumatic valvular heart disease (0.9%), aortic aneurism (0.9%), peripheral artery disease (0.4%) and endocarditis (0.4%) [6].

In this scoping review, specifically in the context of CVD, rheumatic heart disease and endocarditis are not considered because of their infectious aetiology. Arterial hypertension is a risk factor for many cardiovascular diseases and for the purposes of this review is considered as an intermediary disease that leads to CVD.

##### Neurodegenerative diseases

The leading noncommunicable neurodegenerative causes of death are Alzheimer’s disease or dementia (20%), Parkinson’s disease (2.5%), motor neuron diseases (0.4%) and multiple sclerosis (0.2%) [8]. Alzheimer’s disease, vascular dementia, frontotemporal dementia and Lewy body disease will be specifically searched, following the pattern of European dementia prevalence studies [39]. Additionally, because amyotrophic lateral sclerosis is the most common motor neuron disease, it is also included in the search [8, 40, 41].

#### Rationale for context

Public health and clinical settings from any geographical location are being considered. The searches will only consider the period between January 2020 and mid-February 2023 due to time constraints.

#### Rationale for type of evidence

Qualitative studies are not considered since they cannot answer the research question. Editorials and opinion pieces, protocols, and conference abstracts will also be excluded. Clinical practice guidelines are not included since the information they contain should be in the original studies and in reviews on which they are based.

### Pilot study

We did a pilot study to test and refine the search strategies, selection criteria and data extraction sheet as well as to get used to the software—Covidence [42]. The pilot study consisted of selecting from the results of the preliminary search matrix 100 papers in order of best fit to the topic, and 100 papers at random. The team comprised 15 individual reviewers (both in the pilot and final reviews) who met daily to revise, enhance, and reach consensus on the search matrices, criteria, and data extraction sheets.

Regarding the selected databases and the platforms used, we conducted various tests, including PubMed/MEDLINE and Ovid/MEDLINE, as well as Ovid/Embase and Elsevier/Embase. Ultimately, we chose Ovid as the platform for accessing both MEDLINE and Embase, utilizing thesaurus Mesh and EmTrees. We manually translated these thesauri to ensure consistency between them. Given that the review team was spread across the UK and Spain, we centralised the search results within the UK team's access to the Ovid license to ensure consistency. Additionally, using Ovid exclusively for accessing both MEDLINE and Embase streamlined the process and allowed for easier access to preprints, which represent the latest research in this rapidly evolving field.

### Identification of research

#### Sources

The searches are being conducted in MEDLINE via Ovid, Embase via Ovid and Embase preprints via Ovid. We also explored the feasibility of searching in *CDC-Authored Genomics and Precision Health Publications Databases* [43]*.* However, the lack of advanced tools to refine the search, as well as the unavailability of bulk downloading prevented the inclusion of this data source. Nevertheless, a search with 15 records for each disease group showed a full overlap with MEDLINE and/or Embase.

#### Search strategy definition

An initial limited search of MEDLINE via PubMed and Ovid was undertaken to identify relevant papers on the topic. In this step, we identified keytext words in their titles and abstracts, as well as thesaurus terms. The SR-Accelerator, Citationchaser, and Yale Mesh Analyzer tools were used to assist in the construction of the search matrix. With all this information, we developed a full search strategy adapted for each included database and information source, optimised by research librarians.

#### Study evidence selection

The complete search strategies are shown in Additional file 3. The three searches are being conducted in parallel. When performing the search, no limits to the type of study or setting are being applied.

Following each search, all identified citations will be collated and uploaded into Covidence (Veritas Health Innovation, Melbourne, Australia, available at www.covidence.org) with the citation details, and duplicates will be removed.

In the title-abstract and full-text screening phase, the first 10% of the papers will be evaluated by two independent reviewers (accounting for 200 or more papers in absolute numbers in the title-abstract phase). Then, a meeting to discuss discrepancies will lead to adjusting inclusion and exclusion criteria and to acquire consistency between reviewers’ decisions. After that, the full screening of the search results will be performed by a single reviewer. Disagreements that arise between reviewers at each stage of the selection process will be resolved through discussion, or with additional reviewers. We maintain an active forum to facilitate permanent contact among reviewers.

The results of the searches and the study inclusion processes will be reported and presented in a flow diagram following the PRISMA-ScR recommendations [22].

#### Expert consultation

The protocol has been refined after consultation with experts in each field (cancer, CVD, and neurodegenerative diseases) who gave input on the scope of the reviews regarding the diverse biomarkers, risk factors, outcomes, and types of prevention relevant to their fields of expertise. In addition, the search strategies have been peer-reviewed by a network of librarians (PRESS-forum in pressforum.pbworks.com) who kindly provided useful feedback.

### Data extraction

We have developed a draft data extraction sheet, which is included as Additional file 4, based on the JBI recommendations [21]. Data extraction will include citation details, study design, population type, biomarker information (name, type, subtype, clinical utility, use of AI technology), disease (group, specific disease), prevention (primary or secondary, lifestyle if primary prevention), and subjective reviewer observations. The data extraction for all papers will be performed by two reviewers to ensure consistency in the classification of data.

### Data analysis and presentation

The descriptive information about the studies collected in the previous phase will be coded according to predefined categories to allow the elaboration of visual summary maps that can allow readers and researchers to have a quick overview of their main results. As in the previous phases, this process will be carried out with the aid of Covidence.

Therefore, a summary of the extracted data will be presented in tables as well as in static and, especially, through interactive evidence gap maps (EGM) created using EPPI-Mapper [44], an open-access web application developed in 2018 by the Evidence for Policy and Practice Information and Coordinating Centre (EPPI-Centre) and Digital Solution Foundry, in partnership with the Campbell Collaboration, which has become the standard software for producing visual evidence gap maps.

Tables and static maps will be made by using R Studio, which will also be used to clean and prepare the database for its use in EPPI-Mapper by generating two Excel files: one containing the EGM structure (i.e. what will be the columns and rows of the visual table) and coding sets, and another containing the bibliographic references and their codes that reviewers had added. Finally, we will use a Python script to produce a file in JSON format, making it ready for importation into EPPI-Reviewer.

The maps are matrixes with biomarker categories/subcategories defining the rows and diseases serving as columns. They define cells, which contain small squares, each one representing each paper included in it. We will use a code of colours to reflect the study design. There will be also a second sublevel in the columns, depending on the map. Thus, for each group of diseases, we will produce three interactive EGMs: two for primary prevention and one for secondary prevention. For primary prevention, the first map will stratify the data to show whether any or which lifestyle has been considered in each paper in combination with the studied biomarker. The second map for primary prevention and the map for secondary prevention will include, as a second sublevel, the subpopulations in which the biomarker has been used or evaluated, which are disease-specific (i.e. cirrhosis for hepatic cancer) researched. The maps will also include filters that allow users to select records based on additional features, such as the use of artificial intelligence in the content of the papers. Furthermore, the EGM, which will be freely available online, will enable users to view and export selected bibliographic references and their abstracts. An example of these interactive maps with dummy data is provided in Additional file 5.

Finally, we will elaborate on two scientific reports for PROPHET. The main report, which will follow the Preferred Reporting Items for Systematic Reviews and Meta-Analyses extension for Scoping Reviews (PRISMA-ScR) recommendations, will summarise the results of the three scoping reviews, will provide a general and global interpretation of the results and will comment on their implication for the SRIA, and will discuss the limitations of the process. The second report will present the specific methodology for the dynamic maps.

## Discussion

This protocol summarises the procedure to carry out three parallel rapid scoping reviews to provide an overview of the available research and gaps in the literature on biomarkers for personalised primary and secondary prevention for the three most common chronic disease groups: cancer, CVD and neurodegenerative diseases. The result will be a common report for the three scoping reviews and the online publication of interactive evidence gap maps to facilitate data visualisation.

This work will be complemented, in a further step of the PROPHET project, by a subsequent mapping report on the scientific evidence for the clinical utility of biomarkers. Both reports are part of an overall mapping effort to characterise the current knowledge and environment around personalised preventive medicine. In this context, PROPHET will also map personalised prevention research programs, as well as bottlenecks and challenges in the adoption of personalised preventive approaches or in the involvement of citizens, patients, health professionals and policy-makers in personalised prevention. The overall results will contribute to the development of the SRIA concept paper, which will help define future priorities for personalised prevention research in the European Union.

In regard to this protocol, one of the strengths of this approach is that it can be applied in the three scoping reviews. This will improve the consistency and comparability of the results between them, allowing for better leveraging of efforts; it also will facilitate the coordination among the staff conducting the different reviews and will allow them to discuss them together, providing a more global perspective as needed for the SRIA. In addition, the collaboration of researchers with different backgrounds, the inclusion of librarians in the research team, and the specific software tools used have helped us to guarantee the quality of the work and have shortened the time invested in defining the final version of this protocol. Another strength is that we have conducted a pilot study to test and refine the search strategy, selection criteria and data extraction sheet. In addition, the selection of the platform of access to the bibliographic databases has been decided after a previous evaluation process (Ovid-MEDLINE versus PubMed MEDLINE, Ovid-Embase versus Elsevier-Embase, etc.).

Only 10% of the papers will undergo screening by two reviewers, and if time permits, we will conduct kappa statistics to assess reviewer agreement during the screening phases. Additionally, ongoing communication and the exchange and discussion of uncertainties will ensure a high level of consensus in the review process.

The main limitation of this work is the very broad field it covers: personalised prevention in all chronic diseases; however, we have tried to maintain decisions to limit it to the chronic diseases with the greatest impact on the population and in the last 3 years, making a rapid scoping review due to time constraints following recommendations from the Cochrane Rapid Review Methods Group [24]; however, as our aim is to identify gaps in the literature in an area of growing interest (personalisation and prevention), we believe that the records retrieved will provide a solid foundation for evaluating available literature. Additionally, systematic reviews, which may encompass studies predating 2020, have the potential to provide valuable insights beyond the temporal constraints of our search.

Thus, this protocol reflects the decisions set by the PROPHET's timetable, without losing the quality and rigour of the work. In addition, the data extraction phase will be done by two reviewers in 100% of the papers to ensure the consistency of the extracted data. Lastly, extending beyond these three scoping reviews, the primary challenge resides in amalgamating their findings with those from numerous other reviews within the project, ultimately producing a cohesive concept paper in the Strategy Research and Innovation Agenda (SRIA) for the European Union, firmly rooted in evidence-based conclusions.

### Supplementary Information

Additional file 1: Glossary.

Additional file 2: Glossary of biomarkers that may define high risk groups.

Additional file 3: Search strategy.

Additional file 4: Data extraction sheet.

Additional file 5: Example of interactive maps in cancer and primary prevention.
