# Supplementary material for: Biomarkers for personalised prevention of chronic diseases: a common protocol for three rapid scoping reviews
Source: Syst Rev. 2024 Jun 1;13:147. doi: 10.1186/s13643-024-02554-9 (PMC11143646; doi:10.1186/s13643-024-02554-9)
Supplement: Supplementary file 1 — Additional file 1: Glossary. [file 13643_2024_2554_MOESM1_ESM.pdf]

Additional file 1: Glossary

- **Prevention:** reduction of the likelihood of developing a disease, sustaining an injury, or experiencing an unfavorable outcome. This study includes both collective (e.g., population screening) and individual (e.g., individual risk stratification) preventive measures (1).

- **Primary prevention:** activities that are carried out before the appearance of a disease or pathology (1).
- **Secondary prevention:** Individual and community measures to reduce diseases prevalence through early detection and prompt intervention and focuses action on individuals who present the disease in its preclinical phase, with manifestations that are not apparent but that allow their detection. It includes early detection or early diagnosis (2,3).
- **Early detection:** population screening and early clinical detection; both are secondary prevention (2):
    - **Population screening:** it is the practice of early detection of the disease that is actively offered to defined population groups susceptible to presenting the disease that do not have symptoms and have not sought medical help (2).
    - **Early clinical detection:** aimed at people who go to health services for various reasons, including symptoms that could be related to the disease being detected or even expressly demand the practice of the detection test. It reinforces population screening and diagnoses cases that the screening indicates as suspicious (2).
- **Risk:** Probability of a negative or positive event occurring in a specified population during a particular period of time. It is commonly measured in epidemiology and clinical research by the cumulative incidence and incidence proportion (2).
- **Stratification:** it consists of the process of or result of separating a sample population into subsamples according to specified criteria, such as age groups, socioeconomic status, risk groups, etc. (2).
    - **Risk Stratification:** At a clinical level it is the process of assign a risk status to patients to improve care and health outcomes.
- **Personalised prevention:** medical model using characterization of individuals' phenotypes and genotypes (e.g., molecular profiling, medical imaging, lifestyle data) for tailoring the right therapeutic strategy for the right person at the right time, and/or to determine the predisposition to disease and/or to deliver timely and targeted prevention (4).
- **Biomarkers:** A substance, structure, characteristic or process that can be objectively measured as an indicator of normal biological processes, pathogenic processes, or biological responses to a therapeutic intervention or to a particular exposure. They do not measure how a person feels or functions (2) (5). There are many types of biomarkers, this study focuses on biomarkers meet the following criteria.
    - Physiological, molecular, cellular, or imaging indicators. Currently in use or in development.
    - Focus on measuring susceptibility or risk, diagnosis, or prediction. If they allow classify people into at least two risk groups.
- **Implementation level**: biomarkers can be classified according to their implementation status as in development (those that are already being studied in humans, but still not

in real situations; and not previous phases of basic research) or implemented (where the clinical utility can already be seen).

- **Cancer diseases:** Conditions where abnormal cells, in a specific part of the body, divide without control and can invade nearby tissues and produce distant metastasis. Included in the C00-C97 codes of the ICD-10 classification (6) of which only some pathologies will be selected based on their magnitude and severity. Intermediary outcomes prior to disease onset of the included pathologies will also be considered as a proxy of the latter.
- **Cardiovascular diseases:** A type of disease that affects the heart or blood of which only some pathologies will be selected based on their magnitude and severity. Intermediary outcomes prior to disease onset of the included pathologies will also be considered as a proxy of the latter.
- **Neurodegenerative diseases:** Disorders that affect the brain as well as the nerves found throughout the human body and the spinal cord included in the G00-G99 codes of the ICD-10 classification (6) of which only some neurodegenerative pathologies will be selected based on their magnitude and severity. Intermediary outcomes prior to disease onset of the included pathologies will also be considered as a proxy of the latter.

1. Piédrola Gil G, Fernández-Crehuet Navajas J. Medicina preventiva y salud pública. 12ª ed. Barcelona: Elsevier Masson; 2016.

2. Porta M, Greenland S, Hernán M, Silva I dos S, Last JM, International Epidemiological Association, editores. A dictionary of epidemiology. Sixth edition. Oxford: Oxford Univ. Press; 2014. 343 p.

3. Piédrola Gil G, Fernández-Crehuet Navajas J. Medicina preventiva y salud pública. 12ª ed. Barcelona: Elsevier Masson; 2016.

4. Council of European Union. Council conclusions on personalised medicine for patients (2015/C 421/03) [Internet]. Brussels: European Union; 2015 dic. Report No.: (2015/C 421/03). Disponible en: https://eur-lex.europa.eu/legal-content/EN/TXT/PDF/?uri=CELEX:52015XG1217(01)&from=FR

5. FDA-NIH Biomarker Working Group. BEST (Biomarkers, EndpointS, and other Tools) Resource [Internet]. Silver Spring (MD): Food and Drug Administration (US); 2016 [citado 3 de febrero de 2023]. Disponible en: http://www.ncbi.nlm.nih.gov/books/NBK326791/

6. World Health Organization. ICD-10. Version 2019 [Internet]. World Health Organization; [citado 16 de febrero de 2023]. Disponible en: https://icd.who.int/browse10/2019/en
