# Supplementary material for: Biomarkers for personalised prevention of chronic diseases: a common protocol for three rapid scoping reviews
Source: Syst Rev. 2024 Jun 1;13:147. doi: 10.1186/s13643-024-02554-9 (PMC11143646; doi:10.1186/s13643-024-02554-9)
Supplement: Supplementary file 2 — Additional file 2: Glossary of biomarkers that may define high risk groups. [file 13643_2024_2554_MOESM2_ESM.pdf]

Additional file 2: Glossary of biomarkers that may define high risk groups.

Diabetes	plasma glucose, fasting plasma glucose, blood sugar, glycaemia, glycated haemoglobin.
Obesity	BMI, weight.
Hypertension	(high) blood pressure.
Hypercholesterolemia/ dyslipidaemia	high-density lipoprotein (HDL), low-density lipoprotein (LDL), very low-density cholesterol (VLDL), Intermediate-density lipoproteins (IDL), cholesterol, atherosclerosis.
Smoking	Cotinine.
Alcohol consumption	Blood alcohol level, blood/breath alcohol concentration/content (BAC), ethanol level.
HBV, HCV	HBsAg, HBV-DNA, anti-HBV IgG, HCV-RNA, anti-HCV IgG.
HPV	HPV types 16, 18, 31, 33, 35, 39, 45, 51, 52, 56, 58, 59, 66, 68.
Helicobacter pylori	*Helicobacter pylori*, CagA, VacA, VacA 8, VacA 9.
HIV infection	HIV infection, immunodeficiency virus, immune deficiency syndrome (AIDS), CD4 lymphocyte count, CD4 count, CD4+ count, T4 count, T-helper cell count, CD4 percent, HIV viral load.
Kidney disease	Serum Creatinine (SCr), Glomerular Filtration Rate (GFR), Cystatin C (CysC), Kidney Injury molecule 1(KIM-1), Neutrophil gelatinase-associated lipocalin (NGAL), Liver-type fatty acid–binding protein (L-FABP), Uromodulin.
Immunization	Antibodies (IgM, IgG, IgA).
Exercise	Metabolic equivalents (METs).
