# Supplementary material for: Biomarkers for personalised prevention of chronic diseases: a common protocol for three rapid scoping reviews
Source: Syst Rev. 2024 Jun 1;13:147. doi: 10.1186/s13643-024-02554-9 (PMC11143646; doi:10.1186/s13643-024-02554-9)
Supplement: Supplementary file 3 — Additional file 3: Search strategy. [file 13643_2024_2554_MOESM3_ESM.pdf]

Additional file 3: Search strategy

The PCC framework was considered to develop the search strategy, and the following topics were obtained for the search: biomarkers, diseases (CVD, neurodegenerative diseases, and cancer), prevention and personalised. For each of them, the corresponding indexed and free terms were included. Each one of the following tables shows an example for each disease group.

"Block" of terms will be combined following this general strategy:
#1 biomarkers
#2 diseases
#3 prevention
#4 personalised, precision, prediction or predictive
#5 #1 AND #2 AND #3 AND #4
#6 #5 Filters: from 2020-2023

Legend:

Common cells

Specific cells
- Cancer
- Cardiovascular diseases
- Neurodegenerative diseases

Cancer
**OVID MEDLINE**
**BIOMARKERS**
(exp Biological factors/ or ("biological factor*" or biomarker* or "bio* marker*").ti,ab,kw,kf.)
(exp *carbohydrates/ or exp *lipids/ or exp *amino acids/ or exp *proteins/ or exp *vitamins/ or exp *steroids/ or exp *Hormones/ or exp *Enzymes/)
(exp genetic markers/ or exp genotype/ or exp genetics/ or exp genomics or ("genetic marker*" or "genotype" or "germline biomarker*" or "genetic*" or "genomic*" or "epigen*" or "epigenetic mark*").ti,ab,kw,kf.)
(exp Microchip Analytical Procedures/) or (Microarray*).ti,ab,kw,kf.
(exp Metabolomics/ or exp Proteomics/ or ("metabolomic*" or "proteomic*" or "lipidomic*" or "immunomic*" or "nutriomic*" or "transcriptomic*" or "radiomic*").ti,ab,kw,kf.)
(exp Carcinogens / or exp Oncogenes/ or ("carcinogen*" or "oncogen*" or "tumor initiator*" or "tumor promoter" or "tumour initiator*" or "tumour promoter*").ti,ab,kw,kf.)
(exp *"Diagnostic Imaging"/ or (imag*).ti,ab,kw,kf.)

DISEASES
(exp Breast Neoplasms / or ("breast cancer*" or "breast neoplasm*" or "breast carcinoma*" or "breast tumo?r*").ti,ab,kw,kf.)
(exp Lung Neoplasms / or ("lung cancer*" or "lung carcinoma*" or "lung neoplasm*" or "lung tumo?r*").ti,ab,kw,kf.)

(exp Prostatic Neoplasms / or ("prostatic neoplasm*" or "prostatic cancer*" or "prostate cancer*" or "prostate tumo?r*").ti,ab,kw,kf.)
(exp Stomach Neoplasms / or ("stomach neoplasm*" or "gastric cancer*" or "stomach cancer*" or "gastric carcinoma*" or "stomach tumo?r*" or "gastric tumo?r*").ti,ab,kw,kf.)
(exp Colorectal neoplasms/ or ("colorectal neoplasm*" or "colorectal cancer*" or "colorectal tumo?r*" or "colon neoplasm*" or "colon tumo?r*" or "colon cancer*" or "rectal neoplasm*" or "rectal tumo?r*" or "rectal cancer*").ti,ab,kw,kf.)
(exp Uterine Neoplasms / or ("uterine cancer*" or "uterine neoplasm*" or "uterus cancer*" or "uterine tumo?r*").ti,ab,kw,kf.)
(exp Uterine Cervical Neoplasms / or ("cervical cancer*" or "uterine cervical neoplasm*" or "uterine cervix cancer*" or "uterine cervical tumo?r*").ti,ab,kw,kf.)
(exp Urinary Bladder Neoplasms/ or ("bladder cancer*" or "Urothelial carcinoma*" or "Transitional cell carcinoma*" or "urinary bladder neoplasm*" or "urinary bladder tumo?r*").ti,ab,kw,kf.)
(exp Pancreatic Neoplasms/ or ("pancreatic cancer*" or "pancreatic neoplasm*" or "pancreas cancer*" or "pancreas tumo?r*").ti,ab,kw,kf.)
(exp Kidney Neoplasms/ or ("kidney cancer*" or "kidney neoplasm*" or "kidney tumo?r*"").ti,ab,kw,kf.)
(exp Liver Neoplasms/ or (“liver cancer*” or “hepatic cancer*” or “liver tumo?r*” or “liver neoplasm*”).ti,ab,kw,kf.)

**PREVENTION**
(exp Primary Health Care/ or ("public health service*" or "primary health care" or "primary care").ti,ab,kw,kf.)
(exp Primary prevention/ or ("primary disease prevent*" or "preventable disease*").ti,ab,kw,kf.)
(exp Preventive Medicine/ or ("preventive medicine*" or "medical prevent*").ti,ab,kw,kf.)
("protective factor*".ti,ab,kw,kf.)
(susceptibil*.ti,ab,kw,kf.)
(exp *smoking/)
(exp *exercise/ or exp *sedentary behavior/)
(exp *"Diet, food, and nutrition"/ or exp *Overweight/)
(exp *Alcohol Drinking/)
(exp *Type 2 Diabetes Mellitus/)
(exp *HIV/)
(exp*Human Papillomavirus Viruses/)
(exp *Helicobacter pylori/)

(exp *air pollution/)
(exp *Immunization /)
("cancer prevent*" or "cancer vaccin*" or "cancer immuniz*".ti,ab,kw,kf.)
(exp Chemoprevention / or ("chemoprevent*" or "chemoprophylax*").ti,ab,kw,kf.)
(exp Prophylactic Surgical Procedures / or ("surgical prevent*").ti,ab,kw,kf.)
(exp *Community Health Planning/ or ("community setting*" or "health care plan*" or "community health plan*").ti,ab,kw,kf.)
(exp *Preventive health service/ or "preventive health service*".ti,ab,kw,kf.)
(exp *Community Health Center/ or ("community health center*" or "community health centre*" or "community care").ti,ab,kw,kf.)
(exp *Health Education/ or ("health educat*" or "health promot*").ti,ab,kw,kf.)
(exp Mass Screening/ or exp Secondary Prevention/ or ("secondary prevent*" or "screening*").ti,ab,kw,kf.)
(exp Population Surveillance/ or ("population surveillance*" or "population screening*").ti,ab,kw,kf.)
(exp Early Diagnosis/ or ("early diagnos*").ti,ab,kw,kf.)

**PERSONALISED**
(exp Precision medicine/ or ("personalised medicine*" or "precision medicine*" or "predict*" or "individualized medicine*" or "personalised medicine*" or "individualised medicine*").ti,ab,kw,kf.)
(exp Risk assessment/ or exp Risk adjustment/ or ("risk stratifi*" or "risk scor*" or "risk assess*" or "risk adjust*").ti,ab,kw,kf.)
(exp Machine learning/)

CVD
**OVID MEDLINE MESH**
**BIOMARKERS**
(exp Biological factors/ or ("biological factor*" or biomarker* or "bio* marker*").ti,ab,kw,kf.)
(exp *carbohydrates/ or exp *lipids/ or exp *amino acids/ or exp *proteins/ or exp *vitamins/ or exp *steroids/ or exp *Hormones/ or exp *Enzymes/)
(exp genetic markers/ or exp genotype/ or exp genetics/ or exp genomics or ("genetic marker*" or "genotype" or "germline biomarker*" or "genetic*" or "genomic*" or "epigen*" or "epigenetic mark*").ti,ab,kw,kf.)
(exp Microchip Analytical Procedures/) or (Microarray*).ti,ab,kw,kf.
(exp Metabolomics/ or exp Proteomics/ or ("metabolomic*" or "proteomic*" or "lipidomic*" or "immunomic*" or "nutriomic*" or "transcriptomic*" or "radiomic*").ti,ab,kw,kf.)
(exp *Diagnostic Imaging/ or imag*.ti,ab,kw,kf.)

DISEASES
(exp *atrial fibrillation/ or exp *atrial flutter/ or 'atrial fibrillation'.ti,ab,kw,kf.)
(exp *cardiomyopathies/ or exp *myocarditis/ or 'cardiomyopath*'.ti,ab,kw,kf. Or 'myocarditis'.ti,ab.)
exp *myocardial ischemia/ or exp *myocardial infarction/ or 'ischemic heart disease'.ti,ab,kw,kf. or 'ischaemic heart disease'.ti,ab,kw,kf. Or 'heart attack'.ti,ab,kw,kf.
exp *heart valve diseases/ OR ("Nonrheumatic valvular heart" OR "calcific aortic valve" OR "degenerative mitral valve" OR "Nonrheumatic heart").ti,ab,kw,kf.
exp *Peripheral Arterial Disease/ or 'Peripheral Arterial Disease'.ti,ab,kw,kf.
exp *Aortic Aneurysm/ OR "'aortic aneurysm".ti,ab,kw,kf.
exp *stroke/ or exp *brain infarction/ or exp *hemorrhagic stroke/ or exp *ischemic stroke/ or 'stroke'.ti,ab,kw,kf. OR exp *Cerebral Hemorrhage/ OR exp *Subarachnoid Hemorrhage/

PREVENTION
(exp Primary Health Care/ or ("public health service*" or "primary health care" or "primary care").ti,ab,kw,kf.)
(exp Primary prevention/ or ("primary disease prevent*" or "preventable disease*").ti,ab,kw,kf.)
(exp Preventive Medicine/ or ("preventive medicine*" or "medical prevent*").ti,ab,kw,kf.)
("protective factor*".ti,ab,kw,kf.)
(susceptibil*.ti,ab,kw,kf.)

(exp *smoking/)
(exp *exercise/ or exp *sedentary behavior/)
(exp *"Diet, food, and nutrition"/ or exp *Overweight/)
(exp *Alcohol Drinking/)
(exp *Type 2 Diabetes Mellitus/)
exp *renal insufficiency, chronic/ or exp *kidney failure, chronic/
(exp *air pollution/)
((exp *blood pressure/ or exp *hypertension/) or ("blood pressure" or "hypertension" or "high blood pressure").ti,ab,kw,kf.)
(exp *hypercholesterolemia/ OR exp lipid metabolism disorders/ or exp dyslipidemias/ or "dyslipedemia*".ti,ab. OR "dyslipidaemia*".ti,ab,kw,kf. OR "high cholesterol".ti,ab,kw,kf.)
exp hyperglycemia/ or exp *Blood Glucose/ or "high blood glucose".ti,ab,kw,kf.
(exp *Community Health Planning/ or ("community setting*" or "health care plan*" or "community health plan*").ti,ab,kw,kf.)
(exp *Preventive health service/ or "preventive health service*".ti,ab,kw,kf.)
(exp *Community Health Center/ or ("community health center*" or "community health centre*" or "community care").ti,ab,kw,kf.)
(exp *Health Education/ or ("health educat*" or "health promot*").ti,ab,kw,kf.)
(exp Mass Screening/ or exp Secondary Prevention/ or ("secondary prevent*" or "screening*").ti,ab,kw,kf.)
(exp Population Surveillance/ or ("population surveillance*" or "population screening*").ti,ab,kw,kf.)
(exp Early Diagnosis/ or ("early diagnos*").ti,ab,kw,kf.)

**PERSONALISED**
(exp Precision medicine/ or ("personalised medicine*" or "precision medicine*" or "predict*" or "individualized medicine*" or "personalised medicine*" or "individualised medicine*").ti,ab,kw,kf.)
(exp Risk assessment/ or exp Risk adjustment/ or ("risk stratifi*" or "risk scor*" or "risk assess*" or "risk adjust*").ti,ab,kw,kf.)
(exp Machine learning/)

Neurodegeneration
**OVID MEDLINE**
**BIOMARKERS**
(exp Biological factors/ or ("biological factor*" or biomarker* or "bio* marker*").ti,ab,kw,kf.)
(exp *carbohydrates/ or exp *lipids/ or exp *amino acids/ or exp *proteins/ or exp *vitamins/ or exp *steroids/ or exp *Hormones/ or exp *Enzymes/)
(exp genetic markers/ or exp genotype/ or exp genetics/ or exp genomics or ("genetic marker*" or "genotype" or "germline biomarker*" or "genetic*" or "genomic*" or "epigen*" or "epigenetic mark*").ti,ab,kw,kf.)
(exp Microchip Analytical Procedures/ or (Microarray*).ti,ab,kw,kf.)
(exp Metabolomics/ or exp Proteomics/ or ("metabolomic*" or "proteomic*" or "lipidomic*" or "immunomic*" or "nutriomic*" or "transcriptomic*" or "radiomic*").ti,ab,kw,kf.)
((exp neuroglia/ or exp microbiota/) or ("neuroglia" or "microbiota" or "microbiome" or "gut microbiota").ti,ab,kw,kf.)
(exp *"Diagnostic Imaging"/ or (imag*).ti,ab,kw,kf.)

DISEASES
(exp amyotrophic lateral sclerosis/ or ("amyotrophic lateral sclerosis" or "gehrig disease" or "lou gehrig disease").ti,ab,kw,kf.)
(exp parkinson disease/ or ("parkinson" or "secondary parkinson disease" or "secondary parkinsonism").ti,ab,kw,kf.)
(exp alzheimer disease/ or ("Alzheimer disease" or "Alzheimer´s disease" or "alzheimer" or "alzheimer dementia").ti,ab,kw,kf.)
(exp Frontotemporal dementia/ or ("Frontotemporal dementia" or "Wilhelmsen-Lynch disease" or "Frontotemporal Lobe Dementia").ti,ab,kw,kf.)
(exp Lewy body disease/ or ("Dementia with lewy body" or "Diffuse Lewy Body Disease" or "Lewy Body Dementia").ti,ab,kw,kf.)
((exp Multiple sclerosis/) or ("Multiple sclerosis").ti,ab,kw,kf.)
((exp Vascular dementia/) or ("Vascular dementia").ti,ab,kw,kf.)

PREVENTION
(exp Primary Health Care/ or ("public health service*" or "primary health care" or "primary care").ti,ab,kw,kf.)
(exp Primary prevention/ or ("primary disease prevent*" or "preventable disease*").ti,ab,kw,kf.)
(exp Preventive Medicine/ or ("preventive medicine*" or "medical prevent*").ti,ab,kw,kf.)

(("protective factor*").ti,ab,kw,kf.)
(susceptibil*.ti,ab,kw,kf.)
(exp *smoking/)
(exp *exercise/ or exp *sedentary behavior/)
(exp *Diet, food, and nutrition/ or exp *Overweight/)
(exp *Alcohol Drinking/)
((exp *blood pressure/ or exp *hypertension/) or ("blood pressure" or "hypertension" or "high blood pressure").ti,ab,kw,kf.)
(exp *hypercholesterolemia/)
(exp *Type 2 Diabetes Mellitus/)
(exp Early Diagnosis/ or ("early diagnos*").ti,ab,kw,kf.)
(exp Mass Screening/ or exp Secondary Prevention/ or ("secondary prevent*" or "screening*").ti,ab,kw,kf.)
(exp Population Surveillance/ or ("population surveillance*" or "population screening*").ti,ab,kw,kf.)
(exp *Community Health Planning / or ("community setting*" or "health care plan*" or "community health plan*").ti,ab,kw,kf.)
(exp *Preventive health service/ or ("Preventive health service*").ti,ab,kw,kf.)
(exp *Community Health Center/ or ("community health center*" or "community health centre*" or "community care").ti,ab,kw,kf.)
(exp *Health Education / or ("health educat*" or "health promot*").ti,ab,kw,kf.)

**PERSONALISED**
(exp Precision medicine/ or ("personalised medicine*" or "precision medicine*" or "predict*" or "individualized medicine*" or "personalised medicine*" or "individualised medicine*").ti,ab,kw,kf.)
(exp Risk assessment / or exp Risk adjustment / or ("risk stratif" or "risk scor*" or "risk assess*" or "risk adjust*").ti,ab,kw,kf.)
(exp Machine learning/)
