# Supplementary material for: Biomarkers for personalised prevention of chronic diseases: a common protocol for three rapid scoping reviews
Source: Syst Rev. 2024 Jun 1;13:147. doi: 10.1186/s13643-024-02554-9 (PMC11143646; doi:10.1186/s13643-024-02554-9)
Supplement: Supplementary file 4 — Additional file 4: Data extraction sheet. [file 13643_2024_2554_MOESM4_ESM.pdf]

Additional file 4: Data extraction sheet

**CODE**		
**Methods**	Study design	RCT<br>Cohort study<br>Case-control study<br>Systematic review<br>N/P (Not Provided)<br>Other design
	Mendelian Randomization	Has Mendelian randomization been used in the study?
**Biomarker(s)**	Name	Name of the biomarker(s) in text format
	Molecular	**Genetics/Genomics**<br>**Epigenetics/Epigenomics**<br>**Transcriptomics**<br>**Metabolomics**<br>**Proteomics**<br>**Microbiomics/Microbiology**<br>**Biochemistry**<br>**Other molecular biomarker**<br>**N/P**
	Cellular	**Histology** (tissue abnormalities)<br>**Cytology** (cell types)<br>**Other cellular biomarker**<br>**N/P**
	Image	**X-Rays**<br>**Ultrasound** (echography, etc)<br>**CT Scan**<br>**MRI**<br>**Scintigraphy** (Gamma)<br>**Mammography**<br>**Other image biomarker**<br>**N/P**
	Physiological	**Blood Pressure**<br>**Anthropometric measures**<br>**Other physiological biomarker**<br>**N/P**
	Clinical Utility	Does the paper mention the clinical utility of the biomarker? (explicitly)
	AI	Did they use AI technology or methods related to AI? (Deep learning, machine learning, clinical trial simulation, etc.)
	Radiomics	Does the paper mention radiomics?
	Technology	If they used a new technology/wearable to measure the biomarker, specify which one<br>Smart watch<br>Pulse oximetry<br>Infrared cameras<br>Other: _________
**Disease**	Group	CVD<br>Cancer<br>Neurodegenerative
	Sp. Disease	Breast cancer<br>Lung cancer<br>Prostatic cancer<br>Gastric cancer<br>Colorectal cancer<br>Uterine cancer<br>Cervical cancer<br>Urothelial cancer<br>Pancreatic cancer<br>Liver cancer

		Kidney cancer<br>Ischemic heart disease<br>Cardiomyopathy and myocarditis<br>Atrial fibrillation and atrial flutter<br>Aortic aneurysm<br>Nonrheumatic valvular heart disease<br>Peripheral artery disease<br>Stroke<br>Stroke -ischemic stroke<br>Stroke - intracerebral hemorrhage<br>Stroke - subarachnoid hemorrhage<br>ALS<br>Parkinson<br>Alzheimer<br>Frontotemporal dementia<br>Vascular dementia<br>Lewy body disease<br>Multiple sclerosis
**Prevention**	Type	Primary<br>Secondary: Screening, early diagnosis, and early detection<br>N/P
	If Primary: Lifestyle	Smoking<br>Exercise<br>Diet<br>Alcohol<br>Air pollution<br>Immunization<br>Obesity<br>Preventive drugs<br>N/P<br>Other:_____Specify which one
	If Secondary: Risk stratification	Did they stratify by risk?
**Population**	Population type	General: without any other condition or healthy participants. (Included in all disease groups)<br>Smoking (all)<br>Alcohol consumption (all)<br>Family history (all)<br>Diabetes (included in CVD and cancer)<br>Hypertension (included in CVD and neurodegenerative diseases)<br>Cholesterol/Dyslipidaemia (included in CVD)<br>Obesity (included in CVD, cancer, and neurodegenerative diseases)<br>HIV (included in cancer)<br>HPV (included in cancer)<br>Helicobacter pylori (included in cancer)<br>Kidney Failure/CKD (included in CVD)<br>Cirrhosis (included in cancer)<br>Hepatitis Virus B/C/D (included in cancer)<br>Sleep (included in neurodegenerative diseases)<br>Apoe genotype (included in neurodegenerative diseases)<br>Hearing impairment (included in neurodegenerative diseases)<br>N/P
**Observations**		Subjective reviewer observations (if needed)
