# Supplementary material for: Biomarkers for personalised prevention of chronic diseases: a common protocol for three rapid scoping reviews
Source: Syst Rev. 2024 Jun 1;13:147. doi: 10.1186/s13643-024-02554-9 (PMC11143646; doi:10.1186/s13643-024-02554-9)

Additional file 5: Example of interactive maps in cancer and primary prevention

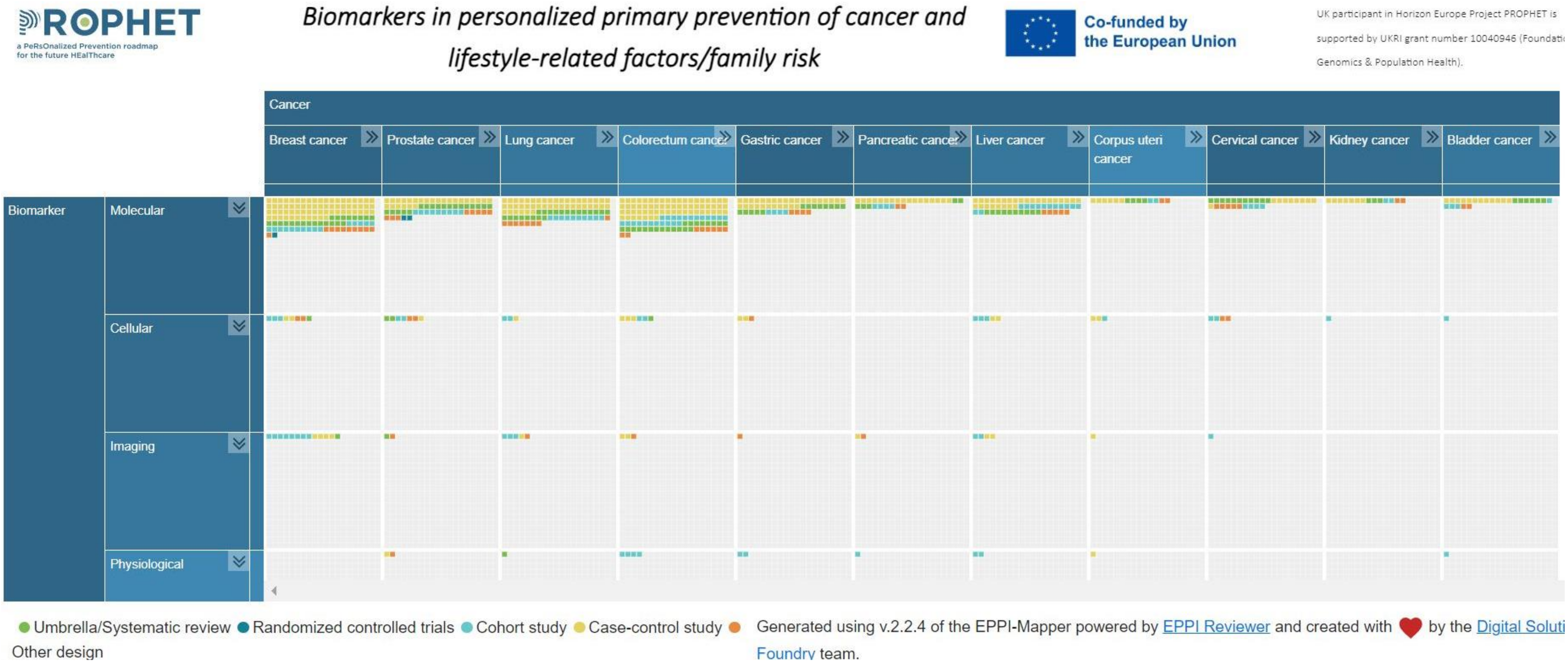

Supplement: Supplementary file 5 — Additional file 5: Example of interactive maps in cancer and primary prevention. [file 13643_2024_2554_MOESM5_ESM.pdf]
